# Psychiatrists’ experiences of suicide assessment

**DOI:** 10.1186/s12888-016-1147-4

**Published:** 2016-12-09

**Authors:** Margda Waern, Niclas Kaiser, Ellinor Salander Renberg

**Affiliations:** 1 Section of Psychiatry and Neurochemistry, Gothenburg University, Blå Stråket 15, 41543 Gothenburg, Sweden; 2 Department of Psychology, Umeå University, Umeå, Sweden; 3 Department of Clinical Sciences, Division of Psychiatry, Umeå University, Umeå, Sweden

**Keywords:** Suicide assessment, Psychiatrists’ experiences, Involuntary care, Medical training, Professional development

## Abstract

**Background:**

Clinical guidelines for suicide prevention often stress the identification of risk and protective factors as well as the evaluation of suicidal intent. However, we know very little about what psychiatrists actually do when they make these assessments. The aim was to investigate psychiatrists’ own accounts of suicide assessment consultations, with a focus on their behaviors, attitudes and emotions.

**Method:**

Semi-structured in depth interviews were carried out with a purposive selection of 15 psychiatrists.

**Results:**

Thematic analysis revealed three main themes: understanding the patient in a precarious situation, understanding one’s own reactions, and understanding how the doctor-patient relationship impacted on risk assessment and management decisions. Emotional contact and credibility issues were common subthemes that arose when the respondents talked about trying to understand the patient. The psychiatrists stressed the semi-intuitive nature of their assessments. Problems related to the use of risk factor assessments and rating scales were apparent. Assessment consultations could evoke physical and emotional symptoms of anxiety, and concerns about responsibility could lead to repressive management decisions. In situations of mutual trust, however, the assessment consultation could kick-start a therapeutic process.

**Conclusion:**

This study highlights psychiatrists’ experiences in clinical suicide assessment situations. Findings have implications for professional development as well as for service delivery.

## Background

WHO member states have agreed to work towards a 10% reduction of suicide rates by the year 2020 [1]. Psychiatric services can play a central role, as persons who seek care in connection with suicidal behavior are at particular risk of suicide [2, 3]. Clinical guidelines for suicide prevention often stress the identification of risk and protective factors as well as the evaluation of suicidal intent [4] and insufficient risk assessment is often highlighted in audits after patient suicide [5].

However, we know very little about what psychiatrists actually do when they assess persons with suicidal issues, and even less about what psychiatrists experience in connection with these consultations. The objective of the study was to investigate psychiatrists’ personal experiences in clinical interactions involving the assessment of suicidal patients.

## Methods

The study is explorative, using a cross-sectional qualitative semi-inductive interview design. The qualitative approach was chosen as this type of study design had the potential to promote disclosure of sensitive material involving the participants’ behaviors, emotions, preferences and attitudes [6, 7] regarding suicide assessments and the clinical decisions that ensue. Our starting point was that psychiatrists would divulge more information in a face-to-face study in which they were given ample opportunity to relate their own experiences, encouraged by a non-judgmental interviewer to “tell more”.

In order to increase richness and variation in the interview data, we approached a purposive sample of psychiatrists based on age, gender, geographic location, and within-psychiatric specialization. Potential participants at three different sites in Sweden (Stockholm, Umeå and Gothenburg) were identified in collaboration with local clinicians. The interviewer then contacted suitable/appropriate participants who fit the profiles that we needed to achieve a purposive sample.

After discussions within the research group it was determined that a total of about 15 interviews would be a functional choice. This number was chosen in relation to the purpose of the study, the study design, the nature of the topic and the anticipated quality of data [8]. Six psychiatrists from Gothenburg, five from Umeå and four from Stockholm were invited to take part in the study; all responded positively. After the 15 interviews were completed it was perceived that we had both sufficient depth and variety enough in our data, a position that sometimes is called saturation. Age, gender and number of years as specialists in psychiatry are shown in Table 1. Thirteen of the participants were employed within adult general psychiatric services; two of these were also employed in emergency psychiatric services, two in addiction medicine and one in forensic psychiatry. One further participant was a geriatric psychiatrist, and one was a psychiatrist working in refugee services. Prior to the interview, all participants received information about the study, and were informed that they would be asked to recall one or two recent clinical situations involving the assessment of persons with suicidal ideation or behavior.

**Table 1:** Participant characteristics^a^ (*N* = 15)

Age group	Gender	Years as specialist
30–34	F	1
35–39	F	1
35–39	M	3
35–39	F	1
35–39	M	7
40–44	F	1
40–44	M	3
40–44	F	8
45–49	M	15
45–49	F	9
50–54	F	18
55–59	F	17
60–64	M	29
65–69	F	28
65-69	M	30

^a^Geographic location and psychiatric subspecialties are shown in text at group level to preserve participant anonymity

The fifteen interviews were carried out during December 2012 to May 2013. The first three were conducted by two of the authors (NK and ESR). The semi-structured interview guide was then evaluated; no significant changes were made. The interview guide is shown in the appendix. Interviews lasted 60–90 min and where held at each clinician’s place of work. They focused on the respondents’ thoughts, feelings and actions during the clinical situations they had chosen to talk about during the interview. The semi-structured interview guide included questions about the specific clinical context, as well as the behaviours of both the doctor and the patient during the assessment process and subsequent management decisions. Interviewees were encouraged to elaborate freely. Towards the end of the interview they were asked specifically about their experiences with suicide assessment scales, and prior to closing they were invited to bring up any relevant information that they felt might have been missed during the interview.

Interviews were audio-recorded, transcribed verbatim and transferred to Open Code Version 4.02 [9] for analysis. The multidisciplinary team used the 6 steps of thematic analysis to identify emergent themes: familiarizing with the data, generating initial codes, searching for, reviewing, defining and naming themes, and producing a report [10]. All authors familiarized with the data independently. Initial coding was conducted by one of the authors (NK). All authors met to discuss codes and identify emergent themes, with specific focus on discrepancies among the researchers’ interpretations. These were discussed until consensus was achieved. Examples of the analysis process are presented in Table 2.

**Table 2:** Examples of the analysis process

Raw text	Condensed meaning units	Code	Category	Theme	Main theme
A consultation that validates the relationship and increases trust is absolutely most important	Validating consultation increases important trust	Validation in assessment leads to trust	Trust and change in the assessment process	Assessment consultation can kick-start a therapeutic process	Understanding the influence of the patient- doctor relationship
Perhaps I crossed the line of what one (the doctor) should or shouldn’t say…..What am I actually doing, when I cross the professional line? ….but at the same time, it is right there, …in the border zone…., that one can make contact and make a change	Doctor on the border on a professional line to make contact and change	Being on the border for contact and change			
	What am I doing when I cross the professional line?	Difficult to understand personal aspects of contact	Personal aspects of the relationship		

## Results

The analysis resulted in a number of subthemes that were organized as three major themes (Table 3), each representing a specific focus for the doctors’ experiences: 1) understanding the patient in a precarious situation, 2) understanding and coping with one’s own feelings, 3) understanding the influence of the patient-doctor interaction.

**Table 3:** Description of themes structure, derived from interviews with psychiatrists (*N* = 15) about suicide assessment

Main themes		
Understanding the patient	Understanding and coping with one’s own reactions	Understanding the influence of the patient- doctor relationship
Subthemes		
Blunted emotional contact hinders assessment	Meeting the suicidal patient elicits emotional reactions	The asymmetrical relationship hinders communication
Credibility issues and the unspoken narrative	Alone and insecure with a deep sense of responsibility	Risking the relationship or risking security?
Impressions elicit gut feelings	Living with uncertainty	Assessment consultation can kick-start a therapeutic process
Not what I learned in school		

### Understanding the patient in a precarious situation

Issues related to emotional contact and credibility were frequently described when the psychiatrists talked about trying to understand their patients. Respondents stressed that the global impression elicited a gut feeling, but this was not something that was taught in medical school. Rating scales were considered unhelpful.

#### Blunted emotion hinders assessment

Most of the respondents brought up the importance of emotional contact. It was difficult to get a grasp on the patient’s motives and intentions when emotional contact was lacking. Assessing risk in a patient with blunted emotions was like flying blind.

*“[I am] able to evaluate better, in a more secure way, when the patient’s story is in contact with a sense of feeling, more substance behind the words. In this case there wasn’t, [a sense of feeling] so it was like flying blind*.”

*“It was a difficult case, extremely difficult, [……], I absolutely did not get good contact with the patient and that makes the assessment so uncertain and poor.”*

Situations in which persons were mild or moderately depressed, with good formal contact but reduced emotional contact could be particularly difficult. One respondent described blunted emotion as a sign of heightened risk.

*[the patient said] “I don’t want to talk to you about this.”….. “blunted contact was a warning signal for me here.”*

Assessment was particularly challenging when substance use issues were involved.

*“(there was) no resonance in the person, one got no feeling of emotion behind the words…(no) eye contact… What does he feel? What does he think? What are his intentions?…the combination (of) lack of emotional contact and substance use, that’s what makes it really precarious.”*

Evaluation of emotional contact was something that the psychiatrist learned over time. Although central to the assessment, it was not something that could be taught during a lecture.

“*Emotional contact is not particularly easy to lecture on or to describe for younger colleagues.*”

Several psychiatrists described situations in which they worked hard to find ways to improve emotional contact during the consultation. When such contact could be achieved, the doctors felt more secure in their suicide assessments.

*“I’m no crystal ball…, but when I get contact, trust develops”*

#### Credibility issues and the unspoken narrative

Getting a grasp on the credibility of the patient’s narrative was frequently described as a central aspect of the assessment procedure.

*“The most important variable is that I perceive him (to be) totally honest in his narrative…..”*

Most of the psychiatrists told of situations that were less than straightforward. The psychiatrists were aware of a hidden agenda. Patients’ narratives were modified depending on the situation; many related situations in which the patient knew what the doctor needed to hear in order to grant ward leave or hospital discharge. One of the informants related how his suicidal patient changed his story when he was assessed by a more senior psychiatrist the day after admission.

*“….he (the patient) was really angry and knew what he needed to say to be released….”*

Another informant described a consultation in which both the doctor and the patient were aware of a parallel narrative.

”*When he understands that I know a bit more he modifies his story, he like accepts the fact* [*that I know more*] *but then he tries a new angle.*”

These situations require tact, because challenging the patient’s credibility could lead to problems further on. But questions needed to be asked.

*“It is alpha and omega, determining the credibility of the patient’s narrative.”*

One of the respondents, however, argued that it was not up to the doctor to question the narrative of the patient.

*” It is not the least difficult to lie to one’s psychiatrist, it is rather easy, it is very easy. It isn’t our job to unveil the patient’s untruths.”*

#### The global impression elicits a gut feeling

Clinical impressions were stressed by respondents. Non-verbal cues were particularly important.

”*Everything is stored somewhere in the brain and in the body and one observes a person’s eye contact, affect, everything, yes, everything merges into a global impression.*”

Respondents described how these clinical impressions translated to a gut feeling, which was essential to the assessment process. After years of experience, the process became semi-intuitive. The gut feeling could help to resolve credibility issues and facilitate management decisions.

*“If you think it through, I don’t know any measure that can give me more, […] the most important thing you have is your gut feeling.”*

While some participants found it difficult to pinpoint what the gut feeling stood for, others tried to make the unexpressed explicit.

*“…eighty percent is in the body language, I don’t know if this is true, but there are subtle ways of saying things, ways of being, ways of looking at one another.”*

*“……. in the end, the gut feeling……. (the patient’s) facial expression, ….. anxiety shining in his eyes, the way of speaking or avoiding a question, those are the things that create security or insecurity (in clinical assessment)*….”.

#### Not what I learned in school

Concern was expressed that relying on the gut feeling might be unprofessional. One participant described a gap between the formal risk assessment approach taught in school and the more intuitive ascertainment of risk. A conflict could arise when there was a lack of congruence between the two.

“*It feels unprofessional […] it is not what I learned in school, where you use risk factors and diverse criteria to make an overall judgment. I do that too, but sometimes there is a conflict between the two and then I have to find out what this feeling stands for and choose which part to act on.”*

Attaining a “real” picture meant that the doctor needed to use implicit and emotional material to draw conclusions related to care and safety. The capability to do this was learned through clinical practice.

*“It’s all about years of practice, practice and more practice.”*

Clinical guidelines called for structured evaluation of risk and protective factors. Some of the informants related how they assessed risk factors in the back of their minds during the consultation, rather than actually ticking off a list or a filling in values on a rating scale.

*“I had it [the list of risk factors] in my head. […] Now wasn’t the right time to pull out a rating scale.*”

Structured risk assessment took time, and time was of the essence.

*”Yes, in liaison psychiatry it is obligatory to do a structured risk assessment but sometimes I choose not to, in order to meet the patient half way, because time is limited, ……I must prioritize what is most important right here and now”.*

Several of the respondents stated openly that they did not follow the clinical guidelines.

“*For a while I tried to be good, […], to note in the patient’s record how many [risk factors] were present, but I think I have lost it a bit […]. I have no sense that rating scales aide my clinical assessment.*”

A couple of the respondents said they used structured instruments regularly. However, a vast majority of the responses to the specific question about suicide assessment scales were negative in character.

*“I don’t think you have any greater use for them (rating scales) in acute situations. It’s more about whether or not the patient trusts me, and dares to tell what he or she is actually thinking and feeling. That’s the foundation.”*

Assessment scales could give the false impression that management decisions were based on objective “evidence.”

*“I don’t believe in ratings scales. Looking at individual risk factors is extremely rigid, and each item doesn’t say much. It is the interactions that provide a basis for clinical assessment. […]. When I am in a bad mood I think that rating scales can only give us a false sense of security”…*

Further, there could be iatrogenic side effects.

*“When one uses ratings scales the consultation is very systematic […] perhaps the essence of reaching a deep and real picture is forgotten, when one asks question after question.”*

This problem could be alleviated by modifying the guidelines’ structured routines, to better fit each individual patient.

*“It mustn’t be too structured or boom, boom, boom […] one has to talk about it and then back off a bit, and then take it up again, … so that they will dare to tell. It’s different, depending on the patient.”*

### Understanding and coping with one’s own feelings

Psychiatrists described experiences of fear, anxiety, and sometimes hopelessness during and after the consultation. A strong sense of responsibility was coupled with feelings of being alone and insecure. Living with uncertainty was part of the job, and respondents described coping strategies that could help in dealing with tough situations.

#### Meeting the suicidal patient elicits emotional and physical reactions

Some of the respondents described feelings of anxiety, including bodily symptoms.

*“He makes me so uncertain and I can say that I felt worry in my gut.”*

*“There is something that bites and digs at my soul, […] a catastrophe is going to happen”*

Helplessness and hopelessness were other feelings that could be elicited during the consultation. One doctor feared that the result of the consultation might be minimal.

*“I solve the acute situation for my own part […] that is, I could say with a clean conscience that I averted a suicidal act, but maybe there wasn’t that much more to it, and perhaps he will take his life in a week’s time.”*

#### Alone and insecure with a deep sense of responsibility

Even though management decisions were sometimes made after a discussion with other mental health professionals, the formal and moral responsibility rested on the doctor. One of the psychiatrists reflected over the fact that not only the patient but also the doctor had loneliness issues.

*“Here we sit, both rather lonely, in different ways…she cannot cope with it, and I must cope, at least with my own vulnerability.”*

Others described feeling worry and frustration, when the patient did not wish the involvement of the psychiatrist.

*“She had no feeling that I could contribute in any way […]. I was not invited to help her or to look at what she needed help with or to explore it together.”*

Some expressed also a moral dimension.

*“I stand with the ultimate responsibility […] to God the Father or Mother, and my decisions can have disastrous consequences for not only the patients but families and society.”*

Several of the respondents meant that when a suicide did occur, the outcome in itself was sufficient to conclude that the doctor must have made an incorrect management decision. One described a situation in which the patient purchased medication on the black market and died of an overdose within a day of the consultation.

*“In some respect I did the wrong thing. I let him go [home] and he went and died.”*

#### Living with uncertainty

The respondents acknowledged the limitations of their assessments, pointing out that uncertainty is a natural part of the psychiatrist’s job.

“*It [the inability to predict] is a part of the essence of prognosis, sometimes it doesn’t happen, and it’s the same for the meteorologist as for the psychiatrist*.”

Some of the respondents related strategies to reduce uncertainty, including talking to colleagues and other care professionals and to persons close to the patient.

*“She [the patient’s mother] was not worried that he would take his life. [She said] “No, no, you can send him home” […]. I asked his mother quite a bit about how he was doing, and it wasn’t that [suicide] she was concerned about, it was his substance use problem that worried her […]. I felt a little bit secure since his mother wasn’t very worried.”*

### Understanding the influence of the patient-doctor interaction

Respondents related problems inherent in the unsymmetrical relationship between patient and doctor. Medicolegal issues led to management decisions that disrupted relationships. A therapeutic alliance could be established already during the assessment, however, when circumstances allowed for an atmosphere of trust.

#### The asymmetrical relationship hinders communication

Several of the psychiatrists brought up the asymmetry of the patient-doctor relationship.

*“She looked at me with her eyes full of fear and said that she was afraid of me.”*

Some psychiatrists told how patients, aware of the asymmetry, might opt not to share information. One described a situation in which the police brought in a middle-aged man, who had been involved in a single car highway accident shortly after discharge from a psychiatric unit where he had been treated with ECT for depression. The first step in the involuntary care process had just been initiated by another doctor, and now it was the psychiatrist’s task to determine whether criteria for involuntary care were fulfilled.

*”Everything he says is to the same end, to show me that he doesn’t have any problems, he doesn’t need to be in the hospital. Everything [the single car accident*] *is a misunderstanding; all he wants is to go home.”*

#### Risking the relationship vs. daring to risk safety

Other psychiatrists described how at times their own uncertainty could lead to a decision to initiate involuntary admission, despite the fact that they were well aware that this could prove counterproductive in the long run. By violating the patient’s freedom and autonomy, compulsory detention would adventure the working alliance. This could even lead to a total break with psychiatric services after discharge.

“*It was this uncertainty, […. the fact that] I could not make a correct assessment […] that was what prompted me to admit him involuntarily. […] Unfortunately this is not the first time that I cause this type of situation […] the patient will have no contact with psychiatric services in the future.”*

The decision not to admit involuntarily was framed in terms of taking a risk, of daring to go along with the patient’s wish to leave the hospital after the consultation. By showing trust, contact was facilitated in the longer run.

“*Well, I’ve been in the situation where I have dared […] sending patients home, and they have come back […] and really started a contact after this.”*

#### Assessment kick-starts the treatment process

When there was emotional contact and the psychiatrist felt more secure, therapeutic strategies including validation and the enhancement of treatment motivation could be applied already during the assessment consultation. The interaction could impart a sense of acknowledgement, and sometimes even instill hope.

*“A consultation that validates the relationship and increases trust is absolutely most important.”*

The application of therapeutic strategies could lead to an intense patient-doctor interaction during the assessment consultation. One of the respondents expressed concern that a violation of professional boundaries might have occurred. At the same time, such an interaction could be of central importance in gaining the patient’s trust, kick-starting the treatment process.

“*Perhaps I crossed the line of what one should or shouldn’t say [during an assessment consultation] […]. What am I actually doing, when I cross the professional line? But at the same time, it is right there, when I’m in the border zone, that things happen […]. I can make contact and make a change.*”

## Discussion

### Findings

Challenges related to blunted emotional contact and credibility issues were common subthemes that arose when the psychiatrists recounted their clinical interactions with suicidal patients. Few mentioned the use of risk factor checklists or rating scales when describing what they actually did during their assessments. The semi-intuitive nature of the assessments was stressed by the respondents. When specifically asked towards the end of the interview about their experiences with suicide rating scales, most reported that they found such instruments unhelpful. Some even considered that the scales could be potentially harmful. An impelling narrative in support of the latter was related in a recent study focusing on the experiences of hospitalized suicidal veterans [11], who meant that rapidly fired questions on the part of the psychiatrist “turned them off and shut them down” (p 165).

Study participants were free to choose the specific encounters they wished to elaborate on during the interview. Many related complex situations involving persons with impulsive behaviours who did not wish to be hospitalized. A feeling that some patients knew exactly what to say in order to avoid inpatient care only increased the doctor’s uncertainty. The psychiatrists reported worries, fears, bodily sensations, frustration and loneliness. None, however described feelings of anger, which might have been anticipated considering findings from previous studies on therapeutic interactions with suicidal patients [12, 13]. It is possible that psychiatrists in our study experienced such feelings but declined to bring up them in the interview, due to concerns about social acceptance or feelings of guilt.

While the psychiatrists were cognizant that they could not prevent all suicides, they experienced a professional, and for some, a moral obligation to keep the patient alive. In a recent Swedish study that examined ethical deliberations among psychiatrists, suicide risk was highlighted as a central issue [14]. Psychiatrists in our study described that the patient-doctor dialogue was often overshadowed by safety issues. They related that management decisions were steered by medicolegal concerns, at times resulting in involuntary detention of persons who were neither deeply depressed not overtly psychotic. Respondents were aware that hospitalization was not always helpful in the long term. The latter was described by service users in Britain who related that a number of stressors had been prompted or exacerbated by psychiatric hospitalization [15].

Some of the respondents reported of collaborative consultations that ended in a positive tone, with the patient starting to regain a sense of control. These situations required that the psychiatrist relinquish some of the control, which could lead to concerns about professional identity, responsibility and patient safety. One of the respondents talked about “crossing the line” for the borders of professionalism. This proved beneficial for the consultation, driving a therapeutic process forward. Vulnerability on the part of the doctor can be appreciated by the patient [16], and might help to create a therapeutic alliance. The alliance has been shown to be associated with better outcomes in a manualized therapeutic intervention for suicidal patients [17], but as of yet little is known about the therapeutic alliance in routine assessment situations.

The gut feeling was a recurring theme in the psychiatrists’ narratives in our study. Clinical decisions were based on a range of impressions evoked by both implicit and emotional information. We know very little about the evidence for such semi-intuitive assessments. We do know that clinical impressions are elicited by a broad range of stimuli that may be difficult to capture using quantitative assessment tools. The latter have thus far yielded disappointing results when it comes to predictive ability [4]. The search for evidence-based models for suicide risk assessment continues, with interesting developments that apply indirect psychological assessments [18], advanced language technology [19], real time in vivo data collection [20], and genomic approaches [21] to improve predictive ability. While such assessment tools may in time prove feasible and reliable in clinical situations, the meeting between the psychiatrist and the suicidal patient remains central. When inpatients treated in connection with suicidal behavior were asked after discharge how services could be improved, they asked for more empathy and compassion from the clinicians they meet [11]. While mental health professionals score lower on stigmatizing attitudes than the general public, social distance is still problematic [22], and users of psychiatric services report stigmatizing experiences in interactions with their professional caregivers [23]. Interventions that support changes in not only attitudes but also behaviors will be required if the (at times) contra productive approaches of today are to be replaced by more collaborative models. The Collaborative Assessment and Management of Suicidality (CAMS) [24], Finn’s therapeutic assessment approach [25] and the Attempted Suicide Short Intervention Programme (ASSIP) [26] are examples of models that are currently gaining attention.

### Methodological considerations

Our study was designed to maximize access to the psychiatrists’ personal experiences and emotions. In line with commonly accepted principles for qualitative methodology, the aim was not to test a specific hypothesis. Nor did the participants constitute a representative sample. A qualitative study involving 15 *other* psychiatrists might yield other results. We recruited through a contact person, which could introduce bias as this person would be likely to identify colleagues who would be positive to taking part in a research study. An alternative approach would be to seek study participants by advertising through the national psychiatric association, but this would introduce another sort of bias. Many psychiatrists do not belong to the association, and among those who do, only those interested in sharing their experiences about suicide assessment would respond to the advertisement.

The use of in-depth individual interviews can be seen as a strength, as psychiatrists might be less candid in a focus group situation. The richness and volume of sensitive information provided by the current study can inform future studies. For example, quantitative questionnaire studies could be designed to test hypotheses about psychiatrists’ consultation styles and emotional responses.

An important limitation is that the current study focuses on the experiences of psychiatrists only; the patients involved in these assessments were not interviewed. A valuable addition for a future study would be to engage patients directly after the initial clinical assessment. Also, insights are needed regarding the experiences of psychologists as well as other mental health professionals including nurses and social workers. Psychiatrists were the focus of this particular study since formal suicide risk assessment is carried out by psychiatrists in the country of Sweden. There are medicolegal reasons for this as decisions about involuntary care due to suicide risk may be made by MDs only.

Reflexivity must be considered in studies that apply qualitative techniques. All three authors are clinical suicidologists with long teaching experience in both university and professional education settings. The idea for the study was generated by the last author, driven by the assumption that psychiatrists’ understanding of suicide assessment could best be captured by descriptions of what they actually do in clinical situations. Years of practice as a liaison psychiatrist in a general hospital setting, the loss of several patients by suicide, as well as the suicide death of a close relative impacted both professionally and personally on the first author. All three authors had a preconception regarding relational aspects of the consultation. This would be expected to impact the way in which the interviewer interacted with the respondents. The coding process was probably not as influenced by preconceptions, as it was conducted using a structured tool, but the group’s discussions about emerging themes was. Credibility and trustworthiness for this study are supported by the detailed description of the research process and the careful outlining of the analysis [27]. Preliminary results have met with a high degree of recognition from both trainees and experienced psychiatrists when presented at educational workshops.

Regarding transferability, all of the psychiatrists were working within the Swedish health care system. The applicability of findings to other settings may vary widely due to geographical and cultural variation in suicidal behaviours [28], as well as differences in assessment and management routines and medicolegal systems. In Sweden, psychiatric services are relatively available and affordable, and one quarter of suicide decedents are in contact with such services during their final year of life [29]. The national suicide rate in Sweden is at a moderately high level (19/100 000), and rates have been basically unchanged since the year 2000 [30]. At the time the interviews were carried out, Swedish legislation required that all suicides that occurred within the context of health care be subject to a national inquiry process.

### Implications

Findings have implications for professional development as well as for service delivery. The accounts of the psychiatrists who took part in this study highlight the importance of interpersonal skills and active listening. Training programs need to support the development of such skills as well as methods to reduce anxiety on the part of psychiatrists. Good role models are important [31]. Suicidal patients want to be listened to by clinicians who are non-judgmental, who allow and invite them to tell their own stories [32]. A person-centred approach that involves service users, their families and care professionals [33] may prove more adaptive in the assessment and care of suicidal patients in the long term, and such models need to be tested.

Some proposals and recommendations for teaching young doctors about the clinical assessment of suicidal patients are listed below.

- Understanding non-verbal signs that may signal increased suicide risk
- Understanding potential advantages and disadvantages involved in the use of risk factor checklists and suicide rating scales
- Improving interpersonal skills to increase psychiatrists’ confidence in talking with patients and their next of kin about suicidal issues
- Improving interviewing techniques to encourage the patient’s narrative
- Learning about how to strengthen the therapeutic alliance during acute consultations
- Involving former patients in workshops to inspire more person-centred approaches

A pertinent question for psychiatric services is how the expectations of the care system in the larger context mesh with the knowledge and attitudes of each individual working within the system. If there is an underlying expectation within the health care system that all suicides among psychiatric patients can be prevented, this may lead to maladaptive responses on the part of the individual clinician [34]. Involuntary care contrasts starkly with concepts of service user involvement and joint decision making, which are stressed in clinical guidelines [35]. Further, a recent large observational study suggested that locked wards do not prevent suicides among psychiatric inpatients [36], pointing to a need for doctors to feel comfortable also when employing less restrictive care practices.

## Conclusion

This study highlights the experiences of psychiatrists in challenging clinical situations involving suicidal persons. Findings suggest that medical and professional training could, in addition to the study of risk and protective factors for suicide, also include a focus on non-verbal communication as well as interpersonal skills to stimulate the patient’s own narrative. Research is needed to determine whether such training programs might facilitate more gratifying clinical experiences for suicidal patients and their doctors alike.

## Abbreviations

ASSIP: Attempted suicide short intervention programme
CAMS: Collaborative assessment of suicidality
